# Non-specific phospholipase C (NPC): an emerging class of phospholipase C in plant growth and development

**DOI:** 10.1007/s10265-020-01199-8

**Published:** 2020-05-05

**Authors:** Yuki Nakamura, Anh H. Ngo

**Affiliations:** Institute of Plant and Microbial Biology, Academia Sinica, 128 sec. 2 Academia Rd., Nankang, Taipei 11529 Taiwan

**Keywords:** Lipid signaling, Non-specific phospholipase C, Phospholipase, Phospholipid

## Abstract

Non-specific phospholipase C (NPC) is a novel class of phospholipase C found only in bacteria and higher plants. NPC hydrolyzes major phospholipid classes such as phosphatidylcholine (PC) and phosphatidylethanolamine (PE) to produce diacylglycerol (DAG) and a corresponding phosphate-containing polar head group. Originally known as a toxin in certain bacteria to invade the host cell, this class of phospholipase has been well-investigated in bacteriology. Since the first discovery of eukaryotic NPC in Arabidopsis in 2005, this emerging class of phospholipase has received greater attention in plant biology in elucidating the biochemical characteristics and physiological function in the context of plant growth regulation and stress response. Particularly in the last few years, there has been significant progress made in understanding the fundamental character of 6 NPC isoforms in Arabidopsis, as well as novel function in other plant models. Now that research with plant NPC is entering into a new phase, this review aims to summarize recent progress in plant NPC along with some future perspectives.

## Introduction

Phospholipase C (PLC) is a class of enzyme that hydrolyzes phospholipids to release diacylglycerol (DAG) and the corresponding polar head group. Phospholipase C is classified into two groups according to the substrate specificity: (1) phosphoinositide-specific PLC (PI-PLC) which specifically hydrolyzes phosphoinositides (PIPs) and (2) non-specific PLC (NPC), also called the phosphatidylcholine-specific phospholipase C (PC-PLC), which is only found in bacteria and plants and non-specifically hydrolyzes major membrane phospholipid classes such as phosphatidylcholine (PC) and phosphatidylethanolamine (PE). The role of PI-PLC is well-characterized in various organisms (Cocco et al. 2015; Pokotylo et al. 2014). For example, animal PI-PLCs are known to activate G protein which is responsible for the regulation of calcium homeostasis. PI-PLC also plays an important role in activating protein kinase C (PKC) as the product DAG binds to PKC (Rhee and Bae 1997). In bacteria, PI-PLC was reported to be a secretory pathogenicity factor (Poussin et al. 2009). In plants, PI-PLCs play a role in plant growth (Zhang et al. 2018a, b) and stress response (Abd-El-Haliem et al. 2016; Kanehara et al. 2015; Xia et al. 2017).

NPC/PC-PLC activity was first detected in a Gram-positive bacteria *Clostridium perfringens* (Macfarlane and Knight. 1941), and later found in *Bacillis cereus* and *Listeria monocytogenes* (reviewed in Titball 1993). PC-PLC was also found in Gram-negative bacteria, such as *Pseudomonas* species (Rossignol et al. 2008), *Burkhoderia pseudomallei* (Korbsrisate et al. 2007) and *Legionella pneumophila* (Aragon et al. 2002). In Gram-positive bacteria, PC-PLC was identified as a potent toxin that is related to *Clostridium perfringens* alpha-toxin. Substrate specificity studies revealed that toxic PC-PLC has a wide range of substrate specificity such as PC, PE, phosphatidylserine (PS) and some other phospholipids (Titball 1993). Toxic bacterial PC-PLCs have been extensively studied for their function as a pathogenicity factor that are responsible for host membrane hydrolysis. Especially, PC-PLCs can also interfere with eukaryotic cellular signaling and take a control of host immune response (Sakurai et al. 2004). In addition, some Gram-positive bacterial PC-PLC possesses functions other than toxicity. For instance, *B. cereus* PC-PLC is involved in the defense mechanism of bacteria to phagocytosis (Rahmet-Alla and Rowley 1990). Thus, some bacterial PC-PLC can be used as vaccines against diseases such as gangrene (Ghannoum 2000). In Gram-negative bacteria, on the other hand, PC-PLCs are non-toxic. Remarkably, these non-toxic type of PC-PLC show sequence similarity to plant NPCs (Nakamura et al. 2005), suggesting that plant NPCs may not be evolutionarily related to toxicity and membrane lysis.

In eukaryotes, the first identification and characterization of NPC date back to 15 years ago in *Arabidopsis thaliana* (Nakamura et al. 2005). Since then, this emerging family of phospholipase has received greater attention in general plant biology research (Nakamura 2014; Pokotylo et al. 2013). In a few recent years, significant progress has been made in understanding the role of uncharacterized isoforms in Arabidopsis as well as emerging physiological functions besides their known roles in stress responses. This review thus aims to provide recent updates on the NPCs, serving as a supplement to the previously published thorough review articles (Nakamura 2014; Pokotylo et al. 2013).

## Emerging functions of NPCs in Arabidopsis

Based on the alignment of amino acid sequences (Nakamura et al. 2005), Arabidopsis NPCs can be classified into two subfamilies according to the presence (NPC1, 2, and 6) or absence (NPC3, 4, and 5) of N-terminal leader sequences. While earlier studies focused on the latter subfamily, it was only in the past few years when physiological function of the first subgroup was revealed. Now that functional characterization is reported for all the isoforms, we herein summarize the biochemical properties and distribution of 6 NPCs evidenced by experimental data, as well as some emerging physiological roles reported in recent years (Table 1).

**Table 1:** Substrate specificity, subcellular localization and biological function of NPCs in Arabidopsis

Genes	Gene locus	Substrate (s)	Subcellular localization	Function	References
*NPC1*	At1g07230	PC	ER, Golgi apparatus	Heat stress response	(Krčková et al. 2015)
*NPC2*	At2g26870	PC, PE	Plastid, ER, Golgi apparatus	Gametophyte development	(Ngo et al. 2018)
				Glycerolipid metabolism	(Ngo et al. 2018)
				Root development	(Ngo et al. 2019)
				Pathogen response	(Krcková et al. 2018)
*NPC3*	At3g03520	LPA	Unknown	Brassinolide signaling, auxin	(Wimalasekera et al. 2010) (Reddy et al. 2010)
*NPC4*	At3g03530	PC, PE	Plasma membrane	Phosphate starvation	(Nakamura et al. 2005) (Wimalasekera et al. 2010)
				Salt stress	(Peters et al. 2010) (Peters et al. 2014) (Kocourková et al. 2011)
				Aluminum stress	(Pejchar et al. 2010) (Pejchar et al. 2015) (Pejchar and Martinec 2015)
				ABA	(Peters et al. 2010) (Kocourková et al. 2011)
				Brassinolide signaling, auxin, cytokinin	(Wimalasekera et al. 2010)
*NPC5*	At3g03540	PC, PE	Cytoplasm, ER	Phosphate starvation	(Gaude et al. 2008)
				Salt stress	(Peters et al. 2014)
*NPC6*	At3g48610	PC, PE, MGDG, DGDG	Plastid, microsomal membrane	Gametophyte development	(Ngo et al. 2018)
				Glycerolipid metabolism	(Ngo et al. 2018)
				Root development	(Ngo et al. 2019)
				Seed yield	(Cai et al. 2020)
				Oil production	(Cai et al. 2020)

*DGDG* digalactosyldiacylglycerol, *ER* endoplasmic reticulum, *ABA* abscisic acid, *LPA* lysophosphatidic acid, *PC* phosphatidylcholine, *PE* phosphatidylethanolamine, *MGDG* monogalactosyldiacylglycerol, *NPC* non-specific phospholipase C

### Biochemical properties

Unlike PI-PLC that exclusively takes PIPs as a substrate, NPC broadly takes major membrane phospholipid classes, such as PC and PE, as a substrate. The recombinant protein of NPC4 and NPC5 showed PLC activity towards PC and PE (Gaude et al. 2008; Nakamura et al. 2005). Similarly, recently cloned NPC2 and NPC6 showed activity to PC and PE with nearly equal substrate preference (Ngo et al. 2018). Also, recombinant NPC1 protein was shown to hydrolyze PC in vitro (Krčková et al. 2015). NPC2, NPC4 and NPC6 were tested for phosphatidic acid (PA) hydrolysis but none of them showed significant phosphatase activity (Nakamura et al. 2005; Ngo et al. 2018), which suggests that NPC works as a phosphodiesterase. However, NPC3 was shown to function as lyso PA phosphatase albeit its high amino acid sequence homology with the other NPCs (Reddy et al. 2010). Of note, rice NPC1 was shown to take PC and non-phosphorus digalactosyldiacylglycerol (DGDG) equally as a good substrate (Cao et al. 2016). Also, a recent report indicated that NPC6 hydrolyzes monogalactosyldiacylglycerol (MGDG) and DGDG (Cai et al. 2020). Thus, further biochemical and enzymological investigation are needed to elucidate the molecular mechanism of substrate specificity.

### Distribution

Tissue expression pattern of 6 Arabidopsis NPCs is reported at transcript level by the analysis of qRT-PCR (Peters et al. 2010; Wimalasekera et al. 2010) and transcriptomic database Genevestigator (data for *NPC*s summerized in Pokotylo et al. 2013). At translational level, GUS reporter assay in different tissues has been reported for NPC3, NPC4 (Wimalasekera et al. 2010), NPC2, and NPC6 (Ngo et al. 2018) but not yet for NPC1 and NPC5. The histochemical observation showed that tissue expression pattern is similar between NPC3 and NPC4 (Wimalasekera et al. 2010), and NPC2 and NPC6 (Ngo et al. 2018). In vegetative tissues, NPC3 and NPC4 are expressed in cotyledon, root tip, and leaf margin. Both NPC2 and NPC6 are preferentially expressed in petioles, leaf vasculature and base of trichome; however, in germinating seedlings, NPC2 is expressed mainly in cotyledon while NPC6 is primarily in hypocotyl. In reproductive organs, these 4 NPCs are highly expressed in pollen. NPC2 is also expressed in stigma and anther filament while NPC6 is expressed mainly in style and ovules.

**Fig. 1:**
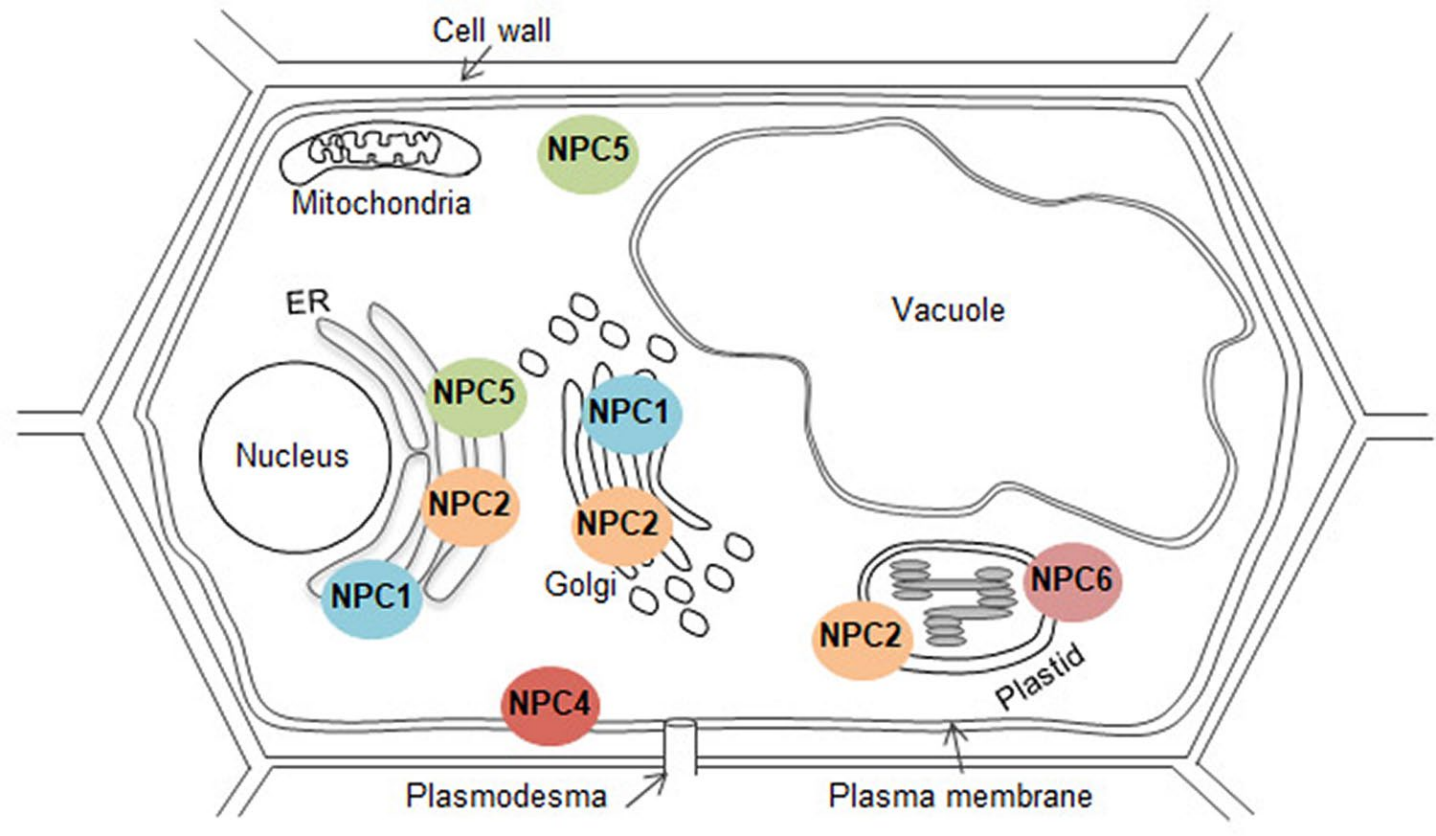
Schematic illustration of the subcellular localization of NPC family in Arabidopsis evidenced by published experimental data

Subcellular localization of 6 NPCs is highly distinct. This was anticipated to some extent because of the presence of N-terminal leader sequence in NPC1, NPC2, and NPC6 but not the others (Nakamura et al. 2005). Based on the reported experimental data, NPC1 is localized at the secretory pathway compartments such as ER, Golgi apparatus, and/or trans-Golgi network in roots (Krčková et al. 2015). NPC2 and NPC6 are localized at chloroplasts in leaf mesophyll cells (Ngo et al. 2018), although mutant phenotypes are unlikely related to chloroplast function (Ngo et al. 2018, 2019). In root, NPC2:GFP fusion protein showed both punctate and reticular structures, which were localized mainly at Golgi apparatus and to a minor extent localized also to the ER and some post-Golgi compartments in the secretory pathway (Krčková et al. 2018). In tobacco leaf mesophyll cells, transiently expressed NPC6-GFP was localized at microsomal membrane as well as chloroplasts (Cai et al. 2020). Thus, these reports suggest that NPC2 and NPC6 might have an alternative subcellular localization depending on tissues. For the rest of NPCs, NPC4 is clearly localized at plasma membranes (Nakamura et al. 2005), whereas NPC5 is mainly present as soluble protein but also in microsomal fraction but not plasma membrane (Gaude et al. 2008). Thus, NPC isoforms are present in distinct subcellular locations and some exhibit dynamic distribution, though at tissue level these isoforms show somewhat overlapping distribution (Fig. 1).

### NPC2 and NPC6 in plant development

Studies on the NPCs so far revealed multiple physiological functions in stressed growth condition; however, the function of NPCs in plant growth and development under normal growth condition has been opened to investigation. A recent study showed that NPC2 and NPC6 have a redundant yet essential role in gametophyte development in Arabidopsis (Ngo et al. 2018). Through the genetic crossing of *NPC1*, *NPC2* and *NPC6*, double homozygous mutant of *npc2 npc6* was shown to be lethal due to a defect in male and female gametophyte development (Ngo et al. 2018). These NPCs are expressed differentially in reproductive organs: whereas both are highly expressed in pollen, NPC6 was more predominantly expressed over NPC2 in ovules. In vitro, NPC2 and NPC6 showed phospholipase C activity to PC and PE but not PA, as was observed with NPC4 (Nakamura et al. 2005). Lipidomic analysis of polar glycerolipid contents revealed that floral buds but not mature flowers of *npc2-1/− npc6-2/* + and *npc2-1/* + *npc6-2/−* increased levels of PC and PE and decreased DGDG amount, so these NPCs may be involved in phospholipid catabolism at the specific stage of flower development. Intriguingly, observation of NPC2 and NPC6 fused with Venus fluorescent marker protein that complemented the lethal phenotype of *npc2-1 npc6-2* showed chloroplast localization in leaf mesophyll cells. Since *npc2-1 npc6-2* double knockout mutant arrests the growth at gametogenesis and hence no viable seeds are obtained, leaky knock-down double mutant lines were created that suppress *NPC2* in *npc6-2* and *NPC6* in *npc2-1* and reproduced gametophyte phenotype of *npc2-1/− npc6-2/* + and *npc2-1/* + *npc6-2/−* to investigate a possible role of NPC2 and NPC6 in vegetative tissues (Ngo et al. 2019). These knock-down mutant lines showed no observed lipid or developmental phenotypes in leaves but reduced root length with increased lateral root density. Interestingly, this reduced root growth phenotype is complemented by supplementation of PCho, a product of NPC-catalyzed reaction. Indeed, these knock-down lines had higher transcript level of *PMT1*, which encodes an enzyme that produces PCho from PEtn and the knock-out mutant shows reduced root length (Cruz-Ramírez et al. 2004). Since the reduced transcript level of *PMT1* was restored to that in wild type following PCho supplementation, NPC2 and NPC6 may interact with PMTs pathway to produce PCho (Ngo et al. 2019), an important factor to promote root growth (Cruz-Ramírez et al. 2004). Thus, NPC2 and NPC6 redundantly play tissue-specific roles in flowers and roots.

### NPC1 in heat stress

Although transcriptomic database indicates that *NPC1* has the highest transcript level among 6 isoforms (Pokotylo et al. 2013), its functional characterization was reported only recently for an important role in plant thermotolerance (Krčková et al. 2015). Loss-of-function mutant *npc1-1* showed an impaired basal thermotolerance while overexpression of NPC1 rendered enhanced resistance to heat stress compared with wild type at high temperature (Krčková et al. 2015). Recombinant NPC1 showed PC-hydrolyzing PLC activity in vitro, and fluorescent reporter assay in root showed that NPC1-GFP was observed in the ER, Golgi apparatus, and/or trans-Golgi network, which suggests that NPC1 is localized at secretory pathway compartments (Krčková et al. 2015). In Tobacco BY-2 cells incubated with fluorescent PC, heat stress increased the production of fluorescent DAG, which suggests that NPC activity is induced by heat stress. These findings provide some hints in understanding a potential role of NPC in heat stress response.

### NPC2 in pathogen attack

Involvement of NPC in biotic stress response is an emerging issue. Based on the transcriptomic database showing that *NPC2* transcript level is downregulated by treatment with elicitor peptide 2, a plant endogenous peptide signal that activates components of the innate immune response (Huffaker et al. 2006; Yamaguchi et al. 2010), qRT-PCR analysis showed that *NPC2* was indeed the most significantly down-regulated among the 6 isoforms after plant infiltration with *Pseudomonas syringae* (Krčková et al. 2018). *NPC* transcript level was decreased also by flagelline peptide flg22 infiltration, expression of the effector molecule AvrRpm1, and salicylic acid (SA) treatment (Krčková et al. 2018). In transgenic plants overexpressing *NPC2*, treatment with flg22 induced higher reactive oxygen species (ROS) production than the wild type, which implies that NPC2 may play a role in response to *Pseudomonas* attack via ROS production (Krčková et al. 2018). As treatment of Arabidopsis protoplast with SA or flg22 decreases the activity to convert fluorescent PC to DAG, NPC2 might be involved in this activity (Krčková et al. 2018).

### NPC4 in aluminum toxicity

Aluminum ion (Al) is a major toxic factor in acidic soils that affects crop production. Using tobacco BY-2 cells incubated with fluorescent PC, AlCl_3_ treatment reduced content of fluorescent DAG, which was more enriched in plasma membrane fraction than in microsomal fraction (Pejchar and Martinec 2015; Pejchar et al. 2010). GUS reporter assay showed that NPC4-GUS expression was reduced after AlCl_3_ treatment in root, and knockout mutant of *NPC4* showed higher sensitivity to AlCl_3_-induced stress than wild type (Pejchar et al. 2015). These findings suggest that NPC4 may be involved in Al toxicity in Arabidopsis.

### NPC5 in salt stress

NPC5 was first characterized for its role in membrane lipid remodeling under phosphate starvation (Gaude et al. 2008). Here, another role of NPC5 was shown in lateral root development under mild salt stress. The *npc5-1* mutant produced fewer lateral root under mild salt stress (75 mM NaCl), while the effect was much weaker at higher or lower NaCl concentrations (Peters et al. 2014). The root phenotype was complemented by transducing full-length genomic *NPC5* into *npc5-1*, and an overexpression of *NPC5* increased lateral root number regardless of salt stress (Peters et al. 2014). NPC5 is induced by salt treatment both at transcriptional and translational levels. Root DAG content was lower in *npc5-1* but higher in *NPC5* overexpression lines, and exogenous supplementation of DAG rescued the root phenotype of *npc5-1* (Peters et al. 2014). Upon treatment of roots with an auxin indole 3-acetic acid (IAA), the increase in the number of lateral roots was less obvious in *npc5-1* than wild type, which resulted in longer length of primary roots (Peters et al. 2014). Thus, NPC5 may be involved in lateral root development under mild salt stress. Aside from NPC5, NPC4 is also known to be involved in salt stress response as *npc4-1* and *npc4-2* mutants showed delay in seed germination and reduction in fresh weight and root length, while overexpression of NPC4 in wild type showed longer root length and increased plant biomass (Kocourková et al. 2011; Peters et al. 2010). A possible interplay between NPC4 and NPC5 regarding salt stress response is open to investigation.

### Rice NPCs: NPC1 in silicon distribution and mechanical strength in stem nodes

In rice (*Oryza sativa*), 5 isoforms of *NPC* (*OsNPC1* to *OsNPC5*) are identified (Singh et al. 2013). Phylogenetic analysis between Arabidopsis and rice NPCs show that AtNPC1, AtNPC2, and AtNPC6 all have a counterpart in rice genome, OsNPC1, OsNPC2, and OsNPC5, respectively. For AtNPC3, 4 and 5, only 2 homologs are present in rice (OsNPC3 and OsNPC4). However, similarity in their amino acid sequences is higher among isoforms within the same species than between species. All the OsNPCs contain highly conserved phosphoesterase domain and the length of their deduced amino acid sequence ranges from 521 to 548 amino acids (Singh et al. 2013). These NPCs show distinct patterns of transcript levels at different developmental stages based on the microarray data. *OsNPC1* reduced the transcript level at early stages of panicle development. *OsNPC1*, *OsNPC2,* and *OsNPC4* were up-regulated towards the end of reproductive development, whereas *OsNPC5* showed rather opposite profile. *OsNPC3* did not show any significant variation in expression during developmental stages (Singh et al. 2013). Later on, qRT-PCR analysis revealed that *OsNPC3* and *OsNPC6* showed the highest transcript level in panicles whereas *OsNPC2* and *OsNPC4* were the highest in roots (Cao et al. 2016). Under salt, cold, and drought stresses, *OsNPC1* to *OsNPC4* but not *OsNPC5* showed increases in transcript level under salt and drought stresses but not cold stress (Singh et al. 2013).

Gene manipulation study is reported only for *OsNPC1* (Cao et al. 2016). Overexpression of *OsNPC1* (*OsNPC1*-OE) produces brittle stem nodes which causes easy bending and increased seed shattering due to easy thresh-off of the head, while RNAi suppression line of *OsNPC1* (OsNPC1-RNAi) showed the opposite effect (Cao et al. 2016). In the node of *OsNPC1*-OE, contents of cellulose, hemicellulose and silicon but not lignin were reduced. The opposite effect was observed in the suppression of *OsNPC1*. Interestingly, OsNPC1 activity isolated from plant hydrolyzes not only phospholipids (PC, PE, phosphatidylglycerol [PG] and PA) but also MGDG and DGDG, with PC and DGDG as the most preferred substrates (Cao et al. 2016). Although it remains elusive how NPC controls silicon distribution in the node, an important agronomic trait for mechanical strength of nodes and grains, a silicon transporter Lsi6 is shown to interact with PA (Cao et al. 2016).

### NPCs in lipid metabolism

In plant biology, phospholipases have been investigated mainly in the context of lipid signaling. Since the lipid signaling involves minor lipid classes such as PA or PIPs, the mutants in phospholipases often show marginal change in membrane lipid content. Whereas it is also the case with some NPC isoforms, such as NPC1 and NPC4 (Krčková et al. 2015; Nakamura et al. 2005), others were shown to be involved in basal phospholipid metabolism as the mutants greatly alter the membrane lipid composition (Gaude et al. 2008; Ngo et al. 2018). The most well-known example is NPC5, which is induced by phosphate starvation and is responsible for the conversion of PC (and possibly some other phospholipid classes) to DGDG (Gaude et al. 2008) in the context of a metabolic conversion called the membrane lipid remodeling (Nakamura 2013). Besides NPC5, recently characterized NPC2 and NPC6 have a redundant role in phospholipid hydrolysis in flower buds, as floral buds of *npc2-1/− npc6-2/* + and *npc2-1/* +  *npc6-2/−* showed increased phospholipid contents (PC, PE, and PG) at the expense of DGDG (Ngo et al. 2018). Since this lipid change was not observed in mature flowers, NPC2 and NPC6 may play a stage-specific role in hydrolyzing phospholipids in floral buds, where lethal defect is observed in the double homozygous mutants (Ngo et al. 2018).

A primary storage lipid triacylglycerol (TAG) is synthesized from DAG that is produced mainly from the turnover of PC (Bates and Browse 2011). Using Arabidopsis cell culture, a similarity in fatty acid composition between PC and TAG and a higher rate of PC to TAG conversion were observed under nitrogen starvation (Mei et al. 2017), a condition that causes massive accumulation of TAG. Transcript levels of *NPC4* and *NPC5* increased in this condition; however, whether any of the NPC mutants alters TAG content remains open to investigation. A very recent report showed that *npc6-1* seeds reduced seed oil contents and weight among the mutants of 6 NPCs (Cai et al. 2020). They further showed that overexpression of NPC6 increased seed oil content and weight in Camelina (*Camelina sativa*) as well as Arabidopsis. Furthermore, a NPC6 homolog in oilseed rape was found to be associated with seed oil content and yield by candidate-gene association study (Cai et al. 2020). These results suggest that NPC6 produces DAG for TAG production.

NPC reaction produces not only DAG but also polar head groups as a byproduct. For example, hydrolysis of PC by NPC produces DAG and phosphocholine (PCho). While the metabolic fate of DAG has received major attention, little is investigated on the role of PCho. Here, a recent report in the transgenic leaky knock-down mutant of *NPC2* in *npc6* knock-out mutant background provided an intriguing insight (Ngo et al. 2019). In addition to the gametophyte-lethal phenotypes similar to *npc2-1/− npc6-2/* + and *npc2-1/* + *npc6-2/−* (Ngo et al. 2018), the knock-down lines showed short root length in seedlings (Ngo et al. 2019). Whereas membrane lipid content was unaffected in the root, exogenous supplementation of PCho rescued the root growth. Interestingly, phospho-base *N*-methyltransferase 1 (PMT1), which encodes an enzyme that produces PCho from phosphoethanolamine, was transcriptionally upregulated in the knockdown line but was attenuated to wild type level upon PCho supplementation (Cruz-Ramírez et al. 2004; Ngo et al. 2019). PMT1 is known to be involved in root growth as the knockout mutant shows considerably short roots (Cruz-Ramírez et al. 2004). Thus, it is possible that PCho produced by NPC activity may have a metabolic interaction with PMT1-mediated methyltransferase pathway and that PCho or its derivative may have a regulatory role in root development.

## Future perspectives

**Fig. 2:**
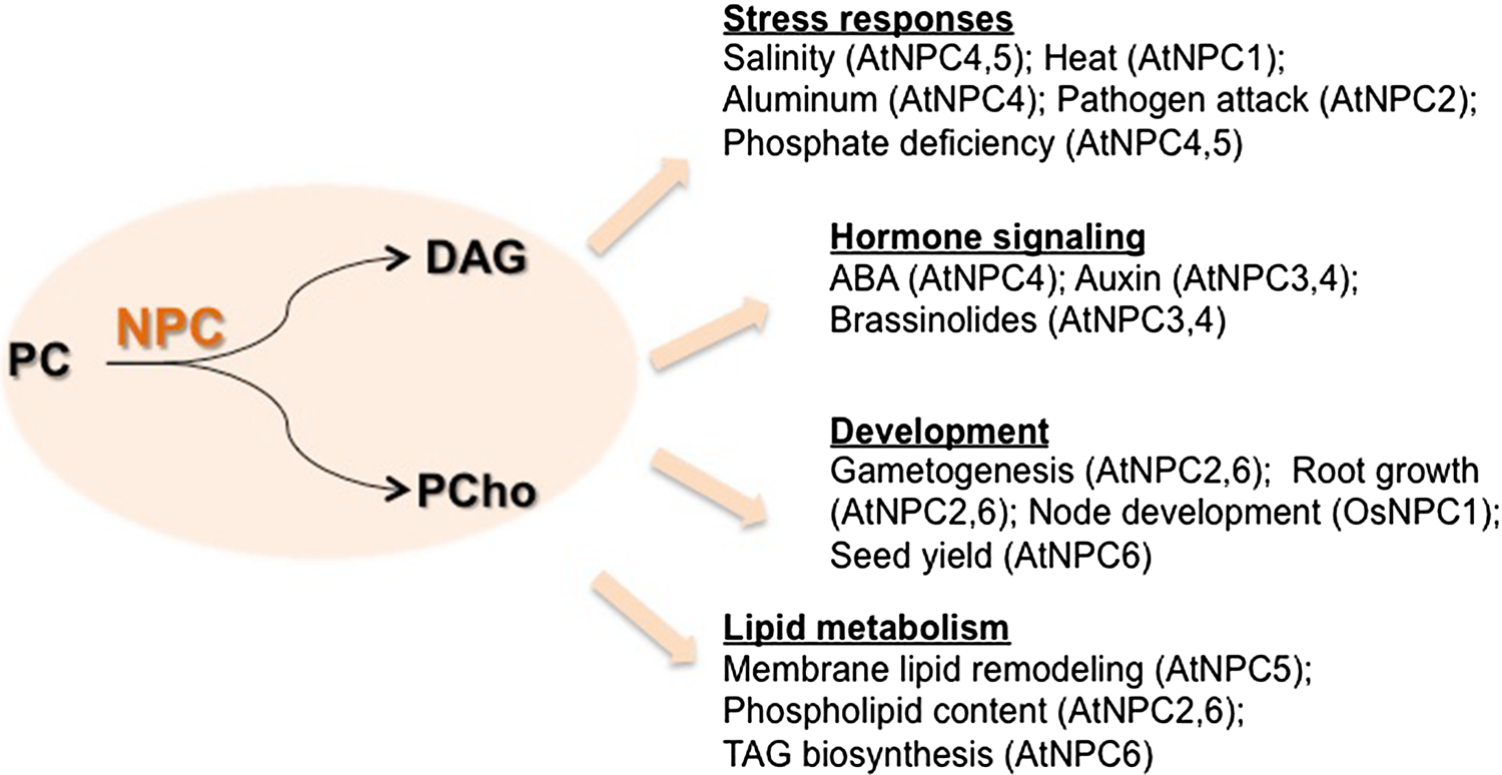
Physiological function of NPC family in plant. *NPC* non-specific phospholipase C, *PC* phosphatidylcholine, *DAG* diacylglycerol, *PCho* phosphocholine, *ABA* abscisic acid, *TAG* triacylglycerol

**Fig. 3:**
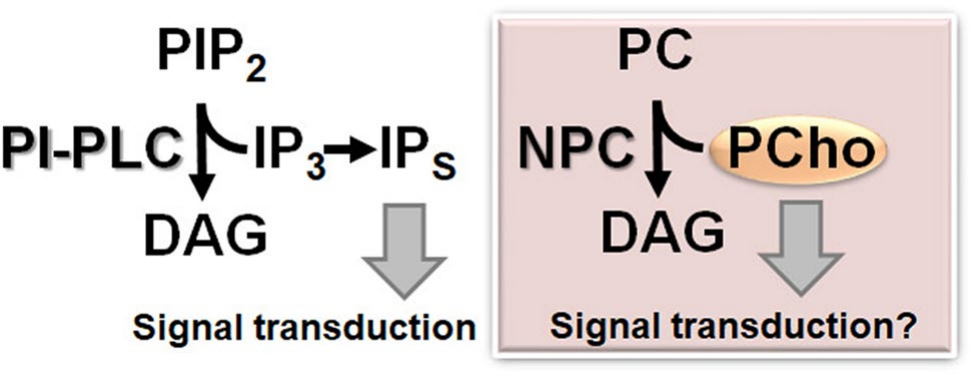
Schematic illustration of reactions catalyzed by PI-PLC and NPC, and a possibility that phosphate-containing product serves as a signaling molecule. PI-PLC, phosphoinositide-specific phospholipase C; IP_3_, inositol 1,4,5-trisphosphate; IPs, inositol polyphosphates. See Fig. 2 legend for the other abbreviations

Now that basic characterization of 6 Arabidopsis NPCs has been reported, NPC research is getting into the next phase. Characterization of mutants and overexpressors showed highly disparate phenotype, which clearly indicates that the NPC family is involved in various aspects of plant growth and development (Fig. 2). Based on the described phenotypes, it is important to address the mechanism by which these NPCs regulate a particular process of growth and development affected in the mutant of respective isoforms. Here, the main focus should be given to the key compound that triggers downstream regulatory cascade. PC hydrolyzed by NPC produces DAG and PCho; however, we have no clear evidence whether either of these products itself functions as a signaling molecule. Unlike in the animal system, it remains unclear whether DAG is a lipid second messenger in plants. Owing to a well-established concept that PA functions as a lipid signal for ample downstream effectors (Pokotylo et al. 2018), it is often assumed that DAG produced by NPC is readily converted to PA by DAG kinase, just in analogy with the DAG production by PI-PLC. However, a clear evidence that NPC-derived DAG is indeed converted to PA for plant function is lacking. On the other hand, a recent report showing that NPC-derived PCho may function in root growth opens up a new idea that the polar head group may also play a regulatory role downstream of NPC reaction. In PI-PLC reaction, both reaction products DAG and IP_3_ have been discussed for regulatory roles. Indeed, IP_3_ is further phosphorylated to produce inositol polyphosphate (IPs) and play important physiological roles in e.g. jasmonate (JA) signaling and phosphate homeostasis (Sheard et al. 2010; Wild et al. 2016). In analogy with this, PCho or its derivative may play a physiological role (Fig. 3). Complementation of short root phenotype in the leaky *NPC2* knock-down in *npc6* knockout mutant by PCho may support this possibility.

Based on the evidence with NPC5 under phosphate starvation and NPC2/NPC6 in floral buds, this phospholipase class may have a significant commitment to primary glycerolipid metabolism. Under phosphate starvation, transcript levels of both *NPC4* and *NPC5* are upregulated (Gaude et al. 2008; Nakamura et al. 2005). Although these NPCs are assumed to take part in the membrane lipid remodeling pathway (Nakamura 2013), how NPC4 contributes to the lipid changes remains unclear because the knockout mutant of NPC4 does not change the lipid contents (Nakamura et al. 2005). A recent work focusing on the interplay between membrane lipid remodeling and JA metabolism under phosphate starvation revealed that *NPC4* transcript level is higher in *coi-1*, a mutant deficient in JA signaling (Chevalier et al. 2019; Xie et al. 1998). This observation implies an involvement of NPC4 in signaling crosstalk rather than primary glycerolipid metabolism. Thus, an important open question is which NPC plays a role in the conversion of PC to DAG in housekeeping glycerolipid metabolism. This reaction is particularly important in two aspects: galactolipid biosynthesis and TAG biosynthesis. In Arabidopsis, galactolipids are synthesized by two pathways with equal contribution; plastid-localized pathway (prokaryotic pathway) and ER-involving pathway (eukaryotic pathway). The PC hypothesis (Roughan 1970) suggests that ER-derived DAG for plastidic galactolipid biosynthesis originates from PC. In TAG biosynthesis, PC to DAG conversion is considered an important reaction step in TAG production (Mei et al. 2017). Although *NPC4* and *NPC5* are transcriptionally upregulated under the condition where TAG is accumulated (Mei et al. 2017), NPC6 plays a role in the context of TAG biosynthesis (Cai et al. 2020). Conversion of PC to DAG can be catalyzed either by NPC or phospholipase D followed by PA phosphatase (Nakamura et al. 2005), so PC to DAG conversion for galactolipid or TAG biosynthesis could be far more complex than thought. Nonetheless, how the 6 NPCs are involved in the biosynthesis of galactolipids and TAG is an important open question to be investigated.

While basic characterizations of all Arabidopsis NPC isoforms are reported, NPC in other plant species are being reported in recent years. Among 5 NPCs in rice, role of NPC1 in silicon distribution at the node was reported (Cao et al. 2016). In cotton, 11 NPC isoforms are reported with distinct gene organization and transcriptional profiles (Song et al. 2017; Zhang et al. 2018a, b, c), albeit functional characterization is yet to be reported. Since the node phenotype with rice NPC1 was not revealed in Arabidopsis *NPC* mutants, NPC might have species-specific roles in regulating plant growth and development. Further investigation on NPC function in major crop species will be an important effort.
