# Correction to: Non-specific phospholipase C (NPC): an emerging class of phospholipase C in plant growth and development

**DOI:** 10.1007/s10265-020-01203-1

**Published:** 2020-05-22

**Authors:** Yuki Nakamura, Anh H. Ngo

**Affiliations:** Institute of Plant and Microbial Biology, Academia Sinica, 128 sec. 2 Academia Rd., Nankang, Taipei 11529 Taiwan

## Correction to: Journal of Plant Research 10.1007/s10265-020-01199-8

In the original publication of the article, values of “Genes” NPC6 was out of order in Table 1. The correct version of Table 1 is provided below.

The original version was updated.

**Table 1:** Substrate specificity, subcellular localization and biological function of NPCs in Arabidopsis

Genes	Gene locus	Substrate (s)	Subcellular localization	Function	References
*NPC1*	At1g07230	PC	ER, Golgi apparatus	Heat stress response	(Krčková et al. 2015)
*NPC2*	At2g26870	PC, PE	Plastid, ER, Golgi apparatus	Gametophyte development	(Ngo et al.2018)
				Glycerolipid metabolism	(Ngo et al.2018)
				Root development	(Ngo et al. 2019)
				Pathogen response	(Krcková et al.2018)
*NPC3*	At3g03520	LPA	Unknown	Brassinolide signaling, auxin	(Wimalasekera et al. 2010) (Reddy et al. 2010)
*NPC4*	At3g03530	PC, PE	Plasma membrane	Phosphate starvation	(Nakamura et al.2005) (Wimalasekera et al. 2010)
				Salt stress	(Peters et al.2010) (Peters et al. 2014) (Kocourková et al. 2011)
				Aluminum stress	(Pejchar et al. 2010) (Pejchar et al. 2015) (Pejchar and Martinec 2015)
				ABA	(Peters et al. 2010) (Kocourková et al. 2011)
				Brassinolide signaling, auxin, cytokinin	(Wimalasekera et al. 2010)
*NPC5*	At3g03540	PC, PE	Cytoplasm, ER	Phosphate starvation	(Gaude et al. 2008)
				Salt stress	(Peters et al. 2014)
*NPC6*	At3g48610	PC, PE, MGDG, DGDG	Plastid, microsomal membrane	Gametophyte development	(Ngo et al. 2018)
				Glycerolipid metabolism	(Ngo et al. 2018)
				Root development	(Ngo et al. 2019)
				Seed yield	(Cai et al. 2020)
				Oil production	(Cai et al. 2020)

*DGDG* digalactosyldiacylglycerol, *ER* endoplasmic reticulum, *ABA* abscisic acid, *LPA* lysophosphatidic acid, *PC* phosphatidylcholine, *PE* phosphatidylethanolamine, *MGDG* monogalactosyldiacylglycerol, *NPC* non-specific phospholipase C

