# Correction to: Non-specific phospholipase C (NPC): an emerging class of phospholipase C in plant growth and development

**DOI:** 10.1007/s10265-020-01251-7

**Published:** 2021-02-05

**Authors:** Yuki Nakamura, Anh H. Ngo

**Affiliations:** Institute of Plant and Microbial Biology, Academia Sinica, 128 sec. 2 Academia Rd., Nankang, Taipei 11529 Taiwan

## Correction to: Journal of Plant Research (2020) 133:489–497 10.1007/s10265-020-01199-8 and 10.1007/s10265-020-01203-1

The article Non-specific phospholipase C (NPC): an emerging class of phospholipase C in plant growth and development, was originally published Online First without Open Access. After publication in volume 133, issue 4, page 489–497 the author decided to opt for Open Choice and to make the article an Open Access publication. Therefore, the copyright of the article has been changed to © The Author(s) 2021 and the article is forthwith distributed under the terms of the Creative Commons Attribution 4.0 International License (https://creativecommons.org/licenses/by/4.0/), which permits use, sharing, adaptation, distribution and reproduction in any medium or format, as long as you give appropriate credit to the original author(s) and the source, provide a link to the Creative Commons license, and indicate if changes were made.

The original article has been updated.

